# Impact of functional capacity on change in self-rated health among older adults in a nine-year longitudinal study

**DOI:** 10.1186/s12877-021-02571-6

**Published:** 2021-11-04

**Authors:** Flávia Silva Arbex Borim, Daniela de Assumpção, Anita Liberalesso Neri, Samila Sathler Tavares Batistoni, Priscila Maria Stolses Bergamo Francisco, Monica Sanches Yassuda

**Affiliations:** 1 Postgraduate Program in Gerontology, School of Medical Sciences, Universidade Estadual de Campinas, Campinas, SP Brazil; 2 Department of Collective Health, School of Health Sciences, University of Brasilia, Brasília, DF Brazil; 3 School of Arts, Sciences and Humanities, Universidade de São Paulo, Arlindo Bettio, 1000, Ermelino Matarazzo, São Paulo, SP CEP 03828-000 Brazil

**Keywords:** Activities of Daily Living (ADLs), Chronic Illness, Depression, Satisfaction, Self-Rated Health

## Abstract

The aims of the present study were to estimate the frequency of change in self-rated health (SRH) among community-dwelling older adults, between two measures taken at a 9-year interval; and determine factors associated with a decline and an improvement in SRH, in relation to aspects of physical/emotional health and subjective wellbeing. Data were derived from a community-based study on frailty among Brazilian elderly. Associations were investigated using Pearson’s chi-square test and relative risk ratios were estimated using multinomial logistic regression analysis. 39.3% of participants did not change their SRH at both assessment times, 21.7% rated it as worse and 39.0% rated it as better. The relative risk ratio of an improvement in SRH for individuals with disability in basic activities of daily living (ADLs) was lower than for individuals with independence in basic ADLs (IRR=0.22; IC95%: 0.08-0.63). Understanding the complex interactions between self-rated health and the dimensions that influence the improvement of health perception may shed light on key determinants of the wellbeing among older adults.

**Supplementary Information:**

The online version contains supplementary material available at 10.1186/s12877-021-02571-6.

## Introduction

Self-rated health (SRH) is a global estimate of one's own health state that generally relies on judgments based on the presence or absence of disease, levels of physical and cognitive capacity, mood and emotional state [1]. Some studies have shown a relation between determinants of wellbeing and positive consequences in terms of social life, economic position and health among older adults [2, 3].

As the very old are susceptible to physical, physiological, psychosocial and cognitive losses, it is expected that their health assessments reflect these losses, becoming more negative than when they were younger. There is some evidence showing that deterioration in physical and mental health and functional performance are related to a decline in SRH [4, 5]. In addition, some research indicates strong associations with socio-economic factors. A study with a statistically representative segment of the Korean older adults investigated the trajectories of self-rated health and observed that different trajectories were related to socioeconomic variables throughout life [6].

Few studies have addressed factors associated to positive change in health assessments, probably due to the rarity of such an occurrence. Verropoulo, in 2012, investigated factors which may predict a decline or an improvement in SRH of older adults and observed that male sex, low educational level and worse health have a strong predictor of subsequent decline in SHR [7]. However, they events are possible and highly relevant as they may signal factors that may have a positive impact in health in aging. Thus, there is the need for a better understanding of the phenomenon of change in SRH and of personal aspects (such as developmental factors) and contextual aspects (such as demographic and socioeconomic characteristics) associated with change in one’s judgment about one’s own health.

The aims of the present study were to estimate the frequency of change in SRH among community-dwelling older adults, between two measures taken at a 9-year interval; and determine factors associated with decline and improvement in SRH, in relation to aspects of physical/emotional health and subjective wellbeing.

## Materials and methods

The data for the present study were derived from the electronic databases of the baseline (2008-2009) and follow-up (2016-2017) investigations of the FIBRA Study, conducted in the city of Campinas (state of São Paulo, Brazil) and the subdistrict of Ermelino Matarazzo, in the city of São Paulo. Both places were chosen by convenience from the probabilistic samples of seven locations of a multicenter study on frailty. The sampling units were the urban census tracts sectors of each city (Fibra Study, 2008-2009) [8].

At baseline, the sample from Campinas comprised 900 older adults and the sample from Ermelino Matarazzo comprised 384, constituting census samples of the population of men and women 65 years of age or older [8]. Recruitment for the follow-up study (2016-2017) was based on the lists of addresses available in the baseline databanks, totaling 1,284 older adults. The recruiters made three attempts to locate each one of the individuals.

**Fig. 1:**
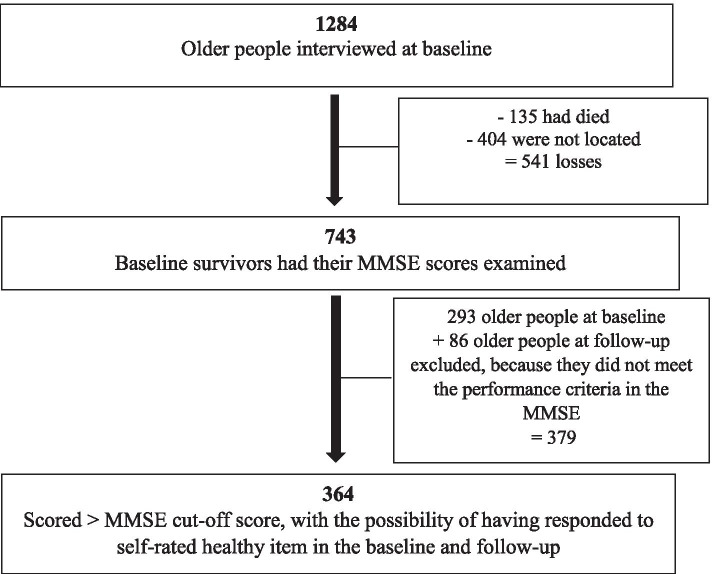
Sample construction decisions and procedures for this investigation. FIBRA study. Legend: MMSE= Mini Mental State Examination

A total of 743 survivors were located and composed the sample to which we applied the eligibility criteria adopted for the FIBRA Study and the present investigation. The exclusion criterion was a score below the education-adjusted cutoff points for the Mini Mental State Examination (MMSE) [9], at baseline and/or follow-up. The cutoff points were 17 for individuals with no formal schooling, 22 for those with one to four years of schooling, 24 for those with five to eight years and 26 for those with nine or more years of schooling [9]. A total of 379 individuals were excluded due to MMSE scores. Among the 364 individuals who met the cognitive criterion and answered the item on self-rated health in the baseline and follow-up were included in the present study (see Figure 1 for the sample flowchart).

### Variables and measures

*Self-rated health (SRH)* was considered the dependent variable and was indicated by the maintenance, decline or improvement in perceived health between baseline and follow-up. At both evaluation times, this variable was measured by the following question: “*In general, how would you rate your health: very good, good, fair, poor or very poor?*”. The variable self-rated health was constructed comparing self-rated health (SRH) at baseline to the one reported at follow-up. More specifically, if the respondent’s SRH at both waves was identical, it was coded as ‘unchanged’ and was assigned the value of 0 (reference category); if SRH at wave 2 had declined it was coded as 1 (for instance, if it had changed from fair to good or very good), while if it had improved it was coded as 2. Hence, a 3-category variable was constructed.

*Multimorbidiy* were indicated by the number of positive answers to nine dichotomous items addressing whether a physician had performed a previous diagnosis of heart disease, systemic arterial hypertension, stroke, diabetes mellitus, cancer, arthritis/rheumatism, depression, lung disease and osteoporosis. Positive answers were counted and the individuals were classified as not having multimorbidity (≤ one disease) or with multimorbidity (≥ two diseases).

*Functional capacity* was measured based on the self-reported ability to perform basic activities of daily living (ADLs). Basic ADLs were investigated using the Katz index ( [10, 11], which addresses the need for help regarding self-care (feeding, continence, transferring, personal hygiene, dressing and taking shower). Individuals who reported requiring partial or complete assistance for carrying out one or more of these activities were considered dependent.

*Depressive symptoms* were evaluated using the 15 dichotomous items of the Geriatric Depression Scale (GDS-15) [12, 13]. The cutoff point adopted for screening for depressive symptoms was > 5.

*Satisfaction with life* was measured using the following question: “*How satisfied are you with your life?*” The answers were low, somewhat and high satisfaction with life.

The following sociodemographic characteristics were used as control variables: sex (self-declared female and male), age range and schooling.

### Data analysis

Descriptive statistics were used for the characterization of the sample based on measures of absolute and relative frequency of the categorical variables, with estimates of percentage distributions and respective 95% confidence intervals, as well as mean and standard deviation values for ordinal variables. Associations between self-rated health and the independent variables (multimorbidity, functional capacity, depressive symptoms and satisfaction with life) and control variables were determined using Pearson's chi-square and Kruskal-Wallis test with a 5% significance level. Relative risk ratios and 95% confidence intervals of the associations between the independent variables and change in SRH were estimated using multinomial logistic regression analysis. Two association models for SRH and the independent variables were tested. Crude relative risk ratios were derived from the first model. In the second model, the relative risk ratios were adjusted by sex, age, schooling, multimorbidity, functional capacity, depressive symptoms and satisfaction with life. Data analysis was performed with aid of the Stata program, version 15.0 (StataCorp, College Station, USA).

## Results

According to the variables studied in the present study, no statistically significant differences were found between the participants and those lost to follow-up.

Among the 364 older adults who composed the final sample, 69.0% were women. At baseline mean age was 71.8 ± 5.09 years; mean years of schooling was 4.5 ± 3.79 years.; 68.0% had two or more chronic diseases and 8.4% reported difficulty performing at least one basic ADLs. Regarding mood, 82.1% did not have symptoms compatible with major depression (GDS > 5), 64.8% were very satisfied with their lives (Table 1).

**Table 1:** Baseline characteristics of 364 individuals aged 65 years and older from the FIBRA Study according to change in self-rated health

Variables	Total ***n***=364	Decline in SRH ***n***=79	Maintenance in SRH ***n***=143	Improvement in SRH ***n***=142	***p***-value
**Sex**					
Male	31.0	22.1	40.7	37.2	0.887
Female	69.0	21.5	38.7	39.8	
**Age, years**					
Mean (±dp)	71.8 (±5.09)	71.7 (±5.24)	72.4 (±5.05)	71.5 (±4.96)	0.421
**Schooling, years**					
Mean (±dp)	4.5 (±3.79)	4.6 (±3.75)	4.9 (±4.34)	4.2 (±3.49)	0.651
**Multimorbidity**					
0 or 1 disease	32.0	28.4	38.8	32.8	0.076
2 or more diseases	68.0	18.6	39.7	41.7	
**Functional capacity**					
Independent	91.6	21.6	36.9	41.5	0.010
Dependent of 1 or more ADLs	8.4	20.0	63.3	16.7	
**Depressive symptoms**					
Without depressive symptoms (GDS≤5)	82.1	21.1	40.1	38.8	0.692
With depressive symptoms (GDS>5)	17.9	23.4	34.4	42.2	
**Satisfaction with life**					
High satisfaction with life	64.8	25.9	38.8	35.3	0.021
More or less satisfaction with life	27.9	15.0	42.0	43.0	
Low satisfaction with life	7.3	7.7	30.8	61.5	

As shown in Table 1, the incidence of improvement in SRH was higher among individuals independent in basic ADLs (independent 40.5% versus dependent 16.7%). The incidence of improvement in SRH was higher among individuals with low satisfaction with life (Table 1).

*SRH* Self-rated Health, *ADLs* Activities of daily living, *GDS* Geriatric Depression Scale

**Fig. 2:**
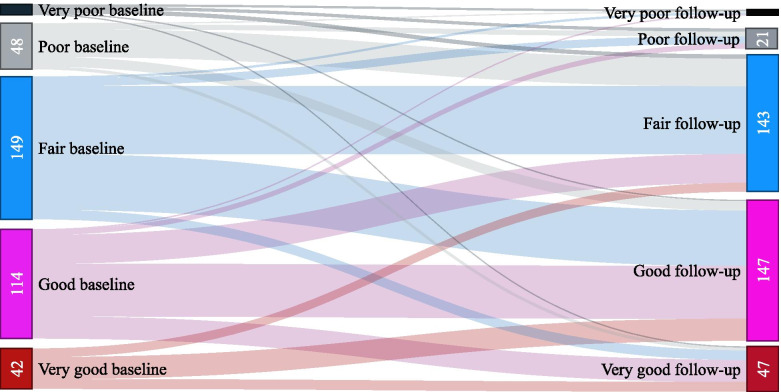
Change in self-rated health among community-dwelling older adults, between two measures taken at a 9-year interval (*n* = 364). FIBRA study

In this sample, 39.3% of the older adults did not show significant change in SRH between both measures, 21.7% rated it as worse and 39.0% rated it as better **(**Table 1). Figure 2 shows the change in SRH among community-dwelling older adults, between two measures taken at a 9-year interval, 34,3% of the older adults showed negative SRH (fair, poor or very poor) on two measures and 12,4% older adults at baseline rated their health as positive and at follow-up changed to negative their SRH.

Table 2 displays the data from the crude and adjusted multinomial logistic regression analyses of associations between the independent variables and SRH. The relative risk ratio of improvement in SRH for individuals with disability basic activities of daily living (ADLs) was lower than for individuals with independence in ADLs (IRR=0.22; IC95%: 0.08-0.63) (Table 2).

**Table 2:** Relative Risk Ratios (RRR) and 95% confidence intervals estimated based on multinomial logistic regression; decline and improvement in self-rated health compared to no change between the waves. FIBRA study

Variables	Crude RRR^a^ (CI95%)^b^	Adjusted RRR^c^ (CI95%)
**Decline in self-rated health**		
0 or 1 disease	1 (Reference)	1 (Reference)
2 or more diseases	0.64 (0.36-1.13)	0.64 (0.35-1.18)
Independent	1 (Reference)	1 (Reference)
Dependent of 1 or more ADLs	0.53 (0.20-1.41)	0.49 (0.18-1.31)
Without depressive symptoms (GDS≤5)	1 (Reference)	1 (Reference)
With depressive symptoms (GDS>5)	1.29 (0.63-2.68)	1.87 (0.83-4.21)
High satisfaction with life	1 (Reference)	1 (Reference)
More or less satisfaction with life	0.53 (0.27-1.05)	0.51 (0.24-1.06)
Low satisfaction with life	0.37 (0,07-1.82)	0.30 (0.06-1.54)
**Improvement in self-rated health**		
0 or 1 disease	1 (Reference)	1 (Reference)
2 or more diseases	1.24 (0.74-2.08)	1.23(0.71-2.12)
Independent	1 (Reference)	1 (Reference)
Dependent of 1 or more ADLs	0.23 (0.08-0.64)*	0.22 (0.08-0.63)*
Without depressive symptoms (GDS≤5)	1 (Reference)	1 (Reference)
With depressive symptoms (GDS>5)	1.27 (0.68-2.36)	1.17 (0.59-2.34)
High satisfaction with life	1 (Reference)	1 (Reference)
More or less satisfaction with life	1.12 (0.66-1.89)	1.09 (0.62-1.92)
Low satisfaction with life	2.19 (0.89-5.39)	1.80 (0.71-4.58)

*ADLs* Activities of daily living, *GDS* Geriatric Depression Scale, ^a^*RRR* Relative Risk Ratios, ^b^*CI95%* 95% Confidence interval, ^c^*Adjusted* Variables adjusted by sex, age, schooling, multimorbidity, functional capacity, depressive symptoms and satisfaction with life

*p<0.05

## Discussion

Although aging and advanced age are commonly associated with worse objective and subjective health indicators, the present study found a substantial proportion (39.0%) of participants who reported improvement in SRH at the follow-up evaluation in comparison to the baseline data collection performed nine years earlier. The individuals with disability in basic ADLs had lower incidence of improvement in SRH than individuals with independence in basic ADLs.

The few studies which have explored changes in disability, perceived health, morbidities and psychological wellbeing reported that if older people improve their physical and mental health, SRH tends to reflect such change [14, 15]. For instance, Vogelsang (2018) [16], based on 21,155 observations in eight waves of data collection among older Americans, found that improvements in SRH were associated with improvement or stability in pre-existing health conditions or the absence of new diagnoses in the more recent waves of data collection. Interestingly, the study also demonstrated that the inclusion of healthy behaviors in one's daily routine (i.e. regular exercise routine), replacing behaviors that posed a health risk, explained the improvement in SRH more than other factors [16].

Present results showed no relation between multimorbidity on objective and subjective health. The term multimorbidity is used to designate the simultaneous presence of two or more chronic diseases in the same individual [17]. Older adults with multimorbidity are more prone to functional disability and polypharmacy and to use healthcare services more often than those with only one chronic disease [17]. Studies have shown that older adults with a negative health assessment have a greater probability of having multimorbidity than those with a positive perception of their health [4, 5]. In the study of Heller et al. (2009), who used the Charlson comorbidity index, an association was observed between changes in comorbidities and decline in SRH in older adults, but the relation was nonlinear and was moderated by age and baseline comorbidity [18].

A previous longitudinal study investigating the bidirectional relation between SRH and physical functioning among older adults found that a negative assessment of one's health was associated with a faster decline in physical functioning [19]. In another longitudinal study, negative SRH was a predictor of disability regarding basic ADL, even after adjusting for confounding variables [20]. In the present study, those who were independent in basic ADL showed an improvement in SRH. Older adults with independence in ADL can engage more frequently in preventive health behaviors and maintain a more active lifestyle, which may lead to an improvement in objective and SRH [21].

No significant association was found between depression symptoms and SRH in the model that included morbidity, functional capacity, and satisfaction with life. Chronic diseases are known to be associated with depression symptoms when mediated by the loss of functional capacity [22]. It is possible that statistical analyses evaluating the mediating function of some variables, such as physical functioning, would have identified more interesting associations. However, the current sample size was too small to allow the use of structural equation modeling.

With the increase in life expectancy and the consequent increase in the longevity of populations, there is greater interest in studies regarding the well-being of older adults, especially when such studies offer data on the satisfaction of social and psychological needs [23]. Some studies have reported a statistically significant association between SRH and subjective well-being [1, 24]. After the adjustment by all independent variables in the present investigation, being very satisfied with life did not increase the relative risk of decline or improvement in SRH. A longitudinal study, that examined the relation between SRH and self-rated economic situation with depressed mood, and life satisfaction as mediator of this relation, among older adults in Costa Rica, observed that the effect of SRH on life satisfaction is less pronounced among those with higher subjective economic situation. Apparently, having a good economic situation can compensate for the impact of poor health on life satisfaction [25].

The present study offered evidence that may strengthen investments in studies of factors related to positive change in aging. We recognize some methodological limitations which may limit the generalization of the results. The sample size may have exerted an influence on some of the associations found between the variables investigated, hindered some stratified analyses and reduced the power of the study. The lack of intermediate assessments between the baseline and the follow-up may also be regarded as a limitation. Moreover, the loss of older adults to follow-up may have incurred in selection bias, as survivors tend to be healthier. Further longitudinal studies should be conducted with larger samples including both adults and older adults to identify differences between these two groups. Moreover, studies should be performed with structural equation modeling, emphasizing not only direct associations but also mediating variables related to change in SRH.

In the present study, improvements in SRH between baseline and follow-up occurred in 39.0% of the older adults. In summary, our main findings suggest that SRH may improve with time among some older adults and independence in basic ADL is an important predictive factor for such an improvement. Understanding the complex interactions between self-rated health and the dimensions that influence change in one's perception of health may shed light on key factors for the wellbeing of older adults and successful aging.

## Supplementary Information

**Additional file 1.**

## Data Availability

The datasets used and/or analyzed during the current study are available from the corresponding author on reasonable request.

## Abbreviations

SRH: Self-rated health
MMSE: Mini Mental State Examination
GDS-15: Geriatric Depression Scale
ADL: Basic Activities of Daily Living
CI 95%: 95% Confidence interval
