# Supplementary material for: Impact of functional capacity on change in self-rated health among older adults in a nine-year longitudinal study
Source: BMC Geriatr. 2021 Nov 4;21:627. doi: 10.1186/s12877-021-02571-6 (PMC8567595; doi:10.1186/s12877-021-02571-6)
Supplement: Supplementary file 1 — Additional file 1. [file 12877_2021_2571_MOESM1_ESM.docx]

| **Variables** | **Participants (364)**  **%** | **Losses**  **(402)**  **%** | **p-value** |
| --- | --- | --- | --- |
| **Sex** |  |  |  |
| Male | 48.1 | 51.9 | p=0.835 |
| Female | 47.3 | 52.7 |  |
| **Age, years** |  |  |  |
| Mean (±DP) | 72.0 (±5.17) | 71.5 (±5.14) | p=0.269 |
| **Schooling, years** |  |  |  |
| Mean (±DP) | 5.1 (±3.94) | 3.8 (±3.46) | p=0.944 |
| **Multimorbidity** |  |  |  |
| 0 or 1 disease | 45.3 | 54.7 | p=0.311 |
| 2 or more diseases | 49.2 | 50.8 |  |
| **Functional Capacity** |  |  |  |
| Independent | 48.2 | 51.8 | p=0.150 |
| Dependent of 1 or more ADLs | 39.5 | 60.5 |  |
| **Depressive symptoms** |  |  |  |
| Without depressive symptoms (GDS≤5) | 47.3 | 52.7 | p=0.930 |
| With depressive symptoms (GDS>5) | 47.8 | 52.2 |  |
| **Satisfaction with life** |  |  |  |
| High satisfaction with life | 48.1 | 51.9 | p=0.871 |
| More or less satisfaction with life | 46.1 | 53.9 |  |
| Low satisfaction with life | 46.4 | 53.6 |  |
| **Self-rated health** |  |  |  |
| very good or good | 45.1 | 54.9 | p=0.221 |
| fair, poor or very poor | 49.5 | 50.5 |  |

Supplementary Material: Comparison between the frequencies of older adults interviewed and lost in the follow-up study, considering the dependent, independent and adjustment variables for this study. FIBRA study.

ADLs: Activities of daily living;

GDS: Geriatric Depression Scale.
